# Comprehensive analysis of oncological outcomes of radical cystectomy for non-muscle invasive bladder cancer

**DOI:** 10.1038/s41598-026-46649-w

**Published:** 2026-04-05

**Authors:** Takeshi Sano, Rikiya Taoka, Jun Miki, Ryoichi Saito, Wataru Fukuokaya, Yoshiyuki Matsui, Shingo Hatakeyama, Takashi Kawahara, Ayumu Matsuda, Taketo Kawai, Minoru Kato, Tomokazu Sazuka, Fumihiko Urabe, Soki Kashima, Hirohito Naito, Yoji Murakami, Makito Miyake, Kei Daizumoto, Yuto Matsushita, Takuji Hayashi, Junichi Inokuchi, Yusuke Sugino, Kenichiro Shiga, Noriya Yamaguchi, Shingo Yamamoto, Keiji Yasue, Takashige Abe, Shotaro Nakanishi, Katsuyoshi Hashine, Masato Fujii, Kiyoaki Nishihara, Hiroaki Matsumoto, Shuichi Tatarano, Koichiro Wada, Sho Sekito, Ryo Maruyama, Naotaka Nishiyama, Hiroyuki Nishiyama, Hiroshi Kitamura, Takashi Kobayashi

**Affiliations:** 1 Department of Urology, Kyoto University Graduate School of Medicine, Kyoto, Japan; 2 Department of Urology and Andrology, Kansai Medical University, Hirakata, Japan; 3 Department of Urology, Faculty of Medicine, Kagawa University, 1750-1 Ikenobe , Kita-gun, Miki-cho, 761-0793 Kagawa Japan; 4 Department of Urology, The Jikei University School of Medicine, Tokyo, Japan; 5 Department of Urology, Jikei University Kashiwa Hospital, Chiba, Japan; 6 Department of Urology, Hyogo Medical University, Nishinomiya, Japan; 7 Department of Urology, National Cancer Center Hospital, Tokyo, Japan; 8 Department of Urology, Hirosaki University Graduate School of Medicine, Hirosaki, Japan; 9 Department of Urology, Faculty of Medicine, University of Tsukuba, Tsukuba, Japan; 10 Department of Urology, Graduate School of Medicine, The University of Tokyo, Tokyo, Japan; 11 Department of Urology, International University of Health and Welfare Ichikawa Hospital, Ichikawa, Japan; 12 Department of Urology, Graduate School of Medicine, Osaka Metropolitan University, Osaka, Japan; 13 Department of Urology, Graduate School of Medicine, Chiba University, Chiba, Japan; 14 Department of Urology, Jikei University Hospital, Tokyo, Japan; 15 Department of Urology, Akita University Graduate School of Medicine, Akita, Japan; 16 Department of Urology, Kurashiki Central Hospital, Kurashiki, Japan; 17 Department of Urology, Graduate School of Life Science, Kumamoto University, Kumamoto, Japan; 18 Department of Urology, Nara Medical University, Kashihara, Japan; 19 Department of Urology, Tokushima University Graduate School of Biomedical Sciences, Tokushima, Japan; 20 Department of Urology, Hamamatsu University School of Medicine, Hamamatsu, Japan; 21 Department of Urology, Osaka International Cancer Institute, Osaka, Japan; 22 Department of Urology, Graduate School of Medical Sciences, Kyushu University, Fukuoka, Japan; 23 Department of Urology, Graduate School of Medicine, University of the Ryukyus, Ginowan, Japan; 24 Department of Nephro-urologic Surgery and Andrology, Mie University Graduate School of Medicine, Tsu, Japan; 25 Department of Urology, Harasanshin General Hospital, Fukuoka, Japan; 26 Department of Urology, Faculty of Medicine, Tottori University, Tottori, Japan; 27 Department of Urology, Jikei University Katsushika Medical Center, Tokyo, Japan; 28 Department of Renal and Genitourinary Surgery, Graduate School of Medicine, Hokkaido University, Sapporo, Japan; 29 Department of Urology, National Hospital Organization Shikoku Cancer Center, Matsuyama, Japan; 30 Department of Urology, Faculty of Medicine, University of Miyazaki, Miyazaki, Japan; 31 Department of Urology, Kurume University School of Medicine, Kurume, Japan; 32 Department of Urology, Graduate School of Medicine, Yamaguchi University, Ube, Japan; 33 Department of Urology, Graduate School of Medical and Dental Sciences, Kagoshima University, Kagoshima, Japan; 34 Department of Urology, Faculty of Medicine, Shimane University, Izumo, Japan; 35 Department of Urology, Aichi Cancer Center Hospital, Nagoya, Japan; 36 Department of Urology, Niigata University Graduate School of Medicine, Niigata, Japan; 37 Department of Urology, Faculty of Medicine, University of Toyama, Toyama, Japan

**Keywords:** Radical cystectomy, Bladder cancer, Non-muscle-invasive bladder cancer, Cancer-specific survival, Neoadjuvant chemotherapy, Lymph node dissection, Cancer, Oncology

## Abstract

Owing to the rarity of radical cystectomy (RC) for non-muscle-invasive bladder cancer (NMIBC), information regarding oncological outcomes is limited. The aim was to conduct a comprehensive analysis of risk factors and survival outcomes following RC. This retrospective study included 435 patients who underwent RC for clinically diagnosed NMIBC at multiple institutions in Japan. The 5-year cancer-specific survival (CSS) rates were 97.4% for cTa, 93.3% for cTis, and 85.4% for cT1, respectively. Among 351 patients with cTa and cT1 disease, multivariable Cox proportional hazards analyses identified upstaging to ≥pT2 or pN+ disease as the most impactful risk factor associated with cancer-specific mortality (hazard ratio = 6.53; *p* < 0.001). Neither neoadjuvant chemotherapy nor lymph node dissection (LND) exhibited a survival benefit in terms of CSS in this cohort; however, LND was occasionally indicative of pN+ disease, suggesting potential diagnostic value. The multivariable logistic regression analysis of preoperative variables identified tumour size (≥ 3 cm) as the lone risk factor associated with upstaging (odds ratio = 1.90; *p* = 0.03). In the sensitivity analyses, tumour size ≥ 3 cm still trended toward an increased upstaging risk, without a statistically significant association. Although the prediction of upstaging remains highly challenging, RC should not be delayed when clinically indicated, even for patients diagnosed with NMIBC, particularly in those with large tumours.

**Supplementary Information:**

The online version contains supplementary material available at 10.1038/s41598-026-46649-w.

## Introduction

Bladder cancer is the tenth most common cancer worldwide, and 573,000 new cases were identified in 2020 alone, 75% of which were diagnosed as non-muscle invasive bladder cancer (NMIBC)^1,2^. Transurethral resection of bladder tumour (TURBT) is the standard treatment for NMIBC, followed by intravesical instillation of chemotherapeutic agents or Bacillus Calmette-Guérin (BCG) as an immunotherapy to prevent recurrence and progression, depending on risk stratification. For carcinoma in situ (CIS), an aggressive form of NMIBC associated with greater risks of recurrence and progression, intravesical instillation of BCG is the standard curative treatment^3–5^. Radical cystectomy (RC) with urinary diversion remains the definitive standard of care for patients with NMIBC who do not respond to or are intolerant of BCG^3–5^, although various bladder-preserving strategies have been explored^6^.

Given the substantial perioperative morbidity associated with RC and its significant impact on patients’ quality of life, the indications for the procedure should be carefully determined. Various guidelines, including those of the European Association of Urology (EAU) and the National Comprehensive Cancer Network (NCCN), have proposed a risk stratification system based on the likelihood of NMIBC recurrence and progression. Within the high-risk category, a subset of “highest-risk” or “very-high-risk” patients have been identified with an especially elevated risk of recurrence and progression for whom early consideration of RC is recommended^4,5^. The Japanese Urological Association (JUA) has also established its own definition of a “very-high-risk group” based on these international guidelines^7^.

Owing to the rarity of RC for NMIBC, the oncological outcomes and risk factors associated with an adverse prognosis are poorly understood. An analysis based on the National Cancer Database bladder dataset from the U.S. over the period of 2006–2016 demonstrated a steady increase in the proportion of patients with NMIBC undergoing RC who received neoadjuvant chemotherapy (NAC), which reached 14.8% in 2016 ^8^. Additionally, retrospective studies of over 1,500 patients with NMIBC who were treated with RC in the U.S. reported that lymph node dissection (LND) was performed in the majority of cases^9^. However, the clinical benefit of NAC or LND in the setting of RC for NMIBC remains uncertain owing to the limited availability of robust evidence, and current clinical guidelines do not offer definitive recommendations regarding their application in RC for NMIBC. A substantial proportion of patients undergoing RC for NMIBC experience pathological upstaging at the time of surgery^10^; in such cases, NAC and LND may be warranted, similar to the management of muscle-invasive bladder cancer (MIBC). However, accurate presurgical identification of patients who are likely to be upstaged at the time of RC remains challenging.

The aim was to conduct a comprehensive analysis of patient characteristics, oncological outcomes, potential roles of NAC and LND, and prognostic factors in a large cohort of patients undergoing RC for NMIBC using data from a Japan Urologic Oncology Group (JUOG)-associated multi-institutional clinical database (JUOG-UC-2021-RC).

## Materials and methods

### Patient selection

**Fig. 1:**
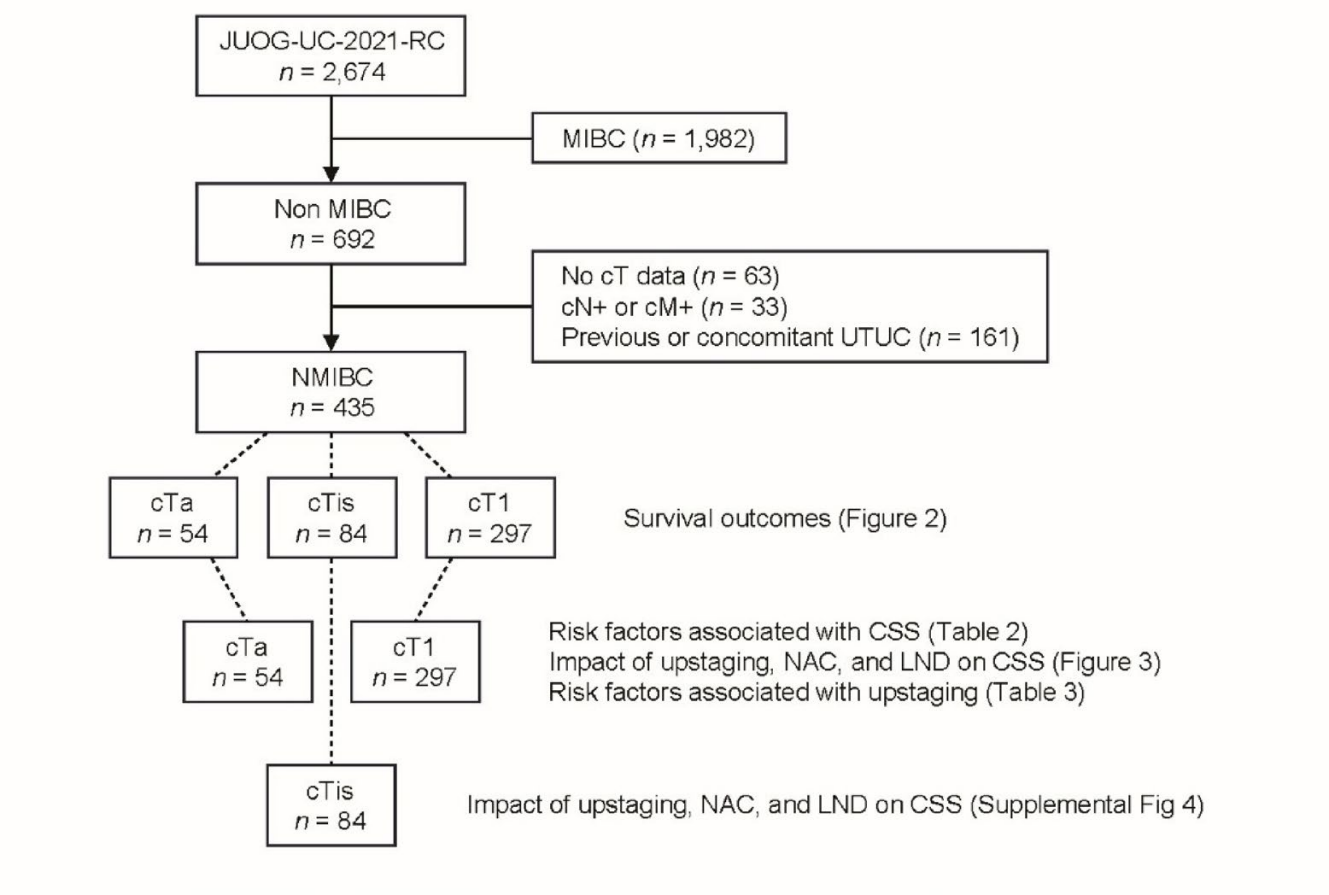
Flowchart summarising the patient exclusion process and the establishment of the analysis cohorts.

After obtaining ethical approval from the Institutional Review Board of Kagawa University (approval number: 2021 − 140), clinical data were retrospectively collected from the medical records of 2,674 consecutive patients diagnosed with bladder cancer who underwent RC between January 2013 and December 2019 at 36 participating institutions affiliated with the JUOG, thereby establishing a Japanese multi-institutional clinical database (JUOG-UC-2021-RC). The cutoff date for follow-up data was December 31st, 2021. Patients were excluded if they were diagnosed with MIBC (*n* = 1,982), lacked data regarding the clinical tumour (T) stage (*n* = 63), presented with lymph node or distant metastasis at the time of RC (*n* = 33), or had a history of upper tract urothelial carcinoma (UTUC) or concurrent UTUC at the time of RC (*n* = 161). Ultimately, 435 patients remained eligible for analysis; however, depending on the purpose of each analysis, specific subsets were excluded, as appropriate (Fig. 1). Details regarding these exclusions are provided in the relevant subsections of the Results. This study follows the principles of the Declaration of Helsinki and its subsequent amendments. Due to the.

retrospective nature of the study, the requirement for informed consent was waived by the Institutional Review Board, with an opt-out method employed.

### Data collection

Among the clinical information collected using a standardised case report form, data related to the following variables were analysed: age; sex; body mass index (BMI); Eastern Cooperative Oncology Group Performance Status (ECOG-PS); smoking status; history of NMIBC and intravesical BCG therapy; tumour multiplicity and size; tumour grade (based on the World Health Organization (WHO) 2004 classification); histological type; clinical and pathological tumour (T) and node (N) stages; perioperative chemotherapy; calendar year of RC; surgical procedures; and extent of LND. Regarding NAC administration, patients were classified into two groups based on the criteria outlined in a report from the JUOG^11^: those who received ≤ 1 cycle were considered to have received insufficient NAC, whereas those who received ≥ 2 cycles were considered to have received sufficient NAC. The extent of LND was categorised as either “none/limited” (defined as no dissection or dissection limited to the obturator nodes) or “standard/extended” (defined as dissection involving the obturator, external iliac, and internal iliac nodes, or beyond). Non-urinary tract recurrence (NUTR) was defined as any recurrence occurring outside the urinary tract, including at pelvic, nodal, or visceral metastatic sites. NUTR was further sub-categorised into intrapelvic recurrence (including pelvic soft-tissue relapse and pelvic lymph node [LN] metastases), distant recurrence (including all extrapelvic metastatic sites), and a combined category when both subclassifications were present.

### Statistical analysis

Descriptive statistics for categorical variables are reported as percentages; for continuous variables, they are presented as medians and interquartile ranges (IQRs). Data are reported in accordance with the Guidelines for Reporting of Statistics for Clinical Research in Urology^12^. Kaplan–Meier analyses were conducted to assess NUTR-free survival (NUTRFS), cancer-specific survival (CSS), and overall survival (OS) based on the date of RC, recurrence or survival events, and the date of such events or the final follow-up, whichever came first. Kaplan–Meier curves were generated using the *survival* and *survminer* packages in R software (version 4.3.1). Intergroup differences in time-to-event outcomes were compared using the log-rank test.

Inverse probability of treatment weighting (IPTW) was conducted using the *WeightIt* package in R. Propensity scores were estimated using logistic regression based on clinically relevant covariates. Stabilised weights were applied, and observations with propensity scores below the 1st percentile or above the 99th percentile were trimmed to reduce the influence of extreme values, thereby generating a weighted pseudo-population in which the distribution of baseline characteristics was balanced between groups. Covariate balance before and after weighting was assessed by calculating standardised mean differences (SMDs) using the *cobalt* package in R, with an SMD < 0.1 considered to be indicative of adequate balance. The covariates included in the propensity score model varied depending on the objective of each analysis and are specified in the corresponding figures displaying the SMDs. Weighted Kaplan–Meier survival curves were generated using the IPTW-adjusted dataset, and intergroup differences were evaluated using a weighted Cox proportional hazards model that incorporated the weights from the IPTW analysis to approximate a marginal structural model. The log-rank test score from the weighted Cox model was used to assess whether statistically significant differences existed between survival curves. The R *survival* package was used to conduct the weighted survival analyses, and the ipw.survival() function was used to generate IPW-adjusted survival curves. For propensity score matching, one-to-one nearest-neighbor matching without replacement was performed with a caliper of 0.2 SD of the logit of the propensity score using the *MatchIt* package in R.

To address instances of missing baseline data for the covariates, the multiple imputation by chained equations method was employed using the *mice* package in R, generating 50 imputed datasets with five iterations per imputation. The imputation models included all of the baseline covariates, exposure variables, and outcome components (including event indicators and log-transformed survival times). All available variables in the dataset were treated as observed covariates and imputed directly using appropriate models (e.g., logistic regression for binary variables and predictive mean matching for continuous variables). Convergence of the multiple imputation process was evaluated by visually inspecting trace plots of the imputed values across iterations, which demonstrated adequate mixing without systematic trends. For each, IPTW based on the propensity score was applied using the *weightit* package, with relevant covariates included in the logistic regression model. A Cox proportional hazards model was subsequently fitted to each weighted dataset to evaluate survival outcomes. The results of the 50 Cox models were pooled using Rubin’s rule to obtain overall hazard ratios (HRs) and confidence intervals (CIs).

Univariable and multivariable analyses were performed using Cox proportional hazards models to estimate HRs for the time to survival events, and logistic regression models were used to estimate odds ratios (ORs) for the prediction of clinical events. Cox models were fitted using the *survival* package. The proportional hazards assumption for all Cox model analyses was assessed using Schoenfeld residuals, and no violations were detected. Logistic regression was performed using the *stats* package preinstalled in Base-R.

## Results

### Study population

The preoperative characteristics of the 435 patients who underwent RC for NMIBC are presented in Table 1. In terms of clinical staging, 54 patients had cTa, 84 had cTis, and 297 had cT1 disease. In the overall cohort, the median age was 70 years (IQR, 65–75 years), 84.6% of the patients were male, only 3.0% had an ECOG-PS ≥ 2, and 57.9% had a history of smoking. Additionally, 42.1% of patients had a history of NMIBC, 37.0% had previously received intravesical BCG therapy, and most patients had high-grade tumours.

**Table 1:** Baseline and perioperative characteristics of patients with cTa, cTis, and cT1 bladder cancer.

	Total		cTa		cTis		cT1	
	(*n* = 435)		(*n* = 54)		(*n* = 84)		(*n* = 297)	
Age at RC, medial (IQR), y	70	(65;75)	71	(65;75)	72	(66;76)	70	(65;75)
Sex, *n* (%)								
Male	368	(84.6)	49	(90.7)	64	(76.2)	255	(85.9)
Female	67	(15.4)	5	(9.3)	20	(23.8)	42	(14.1)
BMI, median (IQR)	23.0	(21.0;25.0)	22.6	(20.8;25.8)	23.0	(20.9;24.9)	23.0	(21.0;24.9)
ECOG PS, *n* (%)								
0 or 1	391	(89.9)	48	(88.9)	74	(88.1)	269	(90.6)
2 or higher	13	(3.0)	0	(0.0)	3	(3.6)	10	(3.4)
Missing	31	(7.1)	6	(1.1)	7	(8.3)	18	(6.1)
Smoking status, *n* (%)								
None	109	(25.1)	14	(25.9)	27	(32.1)	68	(22.9)
Past/current	252	(57.9)	29	(53.7)	41	(48.8)	182	(61.3)
Missing	74	(17.0)	11	(20.4)	16	(19.0)	47	(15.8)
History of NMIBC, *n* (%)								
No	240	(55.2)	21	(38.9)	31	(36.9)	188	(63.3)
Yes	183	(42.1)	31	(57.4)	50	(59.5)	102	(34.3)
Missing	12	(2.8)	2	(3.7)	3	(3.6)	7	(2.4)
History of BCG, *n* (%)								
No	245	(56.3)	23	(51.9)	20	(32.1)	202	(68.0)
Yes	161	(37.0)	25	(37.0)	59	(61.9)	77	(25.9)
Missing	29	(6.7)	6	(11.1)	5	(6.0)	18	(6.1)
Tumour multiplicity, *n* (%)								
Single	87	(24.8)	12	(22.2)	NA		75	(25.3)
Multiple	223	(63.5)	31	(57.4)	NA		192	(64.6)
Missing	41	(11.7)	11	(20.4)	NA		30	(10.1)
Size of tumours, *n* (%)								
Less than 3 cm	194	(55.3)	26	(48.1)	NA		168	(56.6)
3 cm or larger	94	(26.7)	11	(20.4)	NA		83	(27.9)
Missing	63	(18.0)	17	(31.5)	NA		46	(15.5)
Tumour grade at TUR, *n* (%)								
Low grade	16	(3.7)	9	(16.7)	1	(1.2)	6	(2.0)
High grade	362	(83.2)	37	(68.5)	59	(70.2)	266	(89.6)
Missing	57	(13.1)	8	(14.8)	24	(28.6)	25	(8.4)
Histology type at TUR, *n* (%)								
Pure UC	384	(88.3)	53	(98.1)	79	(94.0)	252	(84.8)
Histological subtype	48	(11.1)	1	(1.9)	4	(4.8)	43	(14.5)
Missing	3	(0.7)	0	(0.0)	1	(1.2)	2	(0.7)
Concurrent CIS at TUR, *n* (%)								
No	226	(64.4)	38	(70.4)	NA		188	(63.3)
Yes	115	(32.8)	14	(25.9)	NA		101	(34.0)
Missing	10	(2.8)	2	(3.7)	NA		8	(2.7)
NAC > = 2 cycles, *n* (%)								
No	373	(85.7)	49	(90.7)	78	(92.9)	246	(82.8)
Yes	62	(14.3)	5	(9.3)	6	(7.1)	51	(17.2)
Surgical procedure, *n* (%)								
Open	242	(55.6)	38	(70.4)	43	(51.2)	161	(54.2)
Laparoscopic	132	(30.3)	11	(20.4)	24	(28.6)	97	(32.7)
Robotic	61	(14.0)	5	(9.3)	17	(20.2)	39	(13.1)
Extent of LND, *n* (%)								
None	39	(9.0)	6	(11.1)	14	(16.7)	19	(6.4)
Limited	33	(7.6)	1	(1.9)	8	(9.5)	24	(8.1)
Standard	202	(46.4)	21	(38.9)	37	(44.0)	144	(48.5)
Extended	154	(35.4)	24	(44.4)	25	(29.8)	105	(35.4)
Missing	7	(1.6)	2	(3.7)	0	(0.0)	5	(1.7)
Pathological T stage, *n* (%)								
T0	80	(18.4)	5	(9.3)	15	(17.9)	60	(20.2)
Ta/Tis/T1	252	(57.9)	34	(63.0)	61	(72.6)	157	(52.9)
T2	55	(12.6)	9	(16.7)	6	(7.1)	40	(13.5)
T3 or greater	46	(10.6)	6	(11.1)	2	(2.4)	38	(12.8)
Missing	2	(0.5)	0	(0.0)	0	(0.0)	2	(0.7)
Pathological N status, *n* (%)								
N0	369	(84.8)	45	(83.3)	69	(82.1)	255	(85.9)
N+	28	(6.4)	4	(7.4)	1	(1.2)	23	(7.7)
NX	38	(8.7)	5	(9.3)	14	(16.7)	19	(6.4)
Adjuvant chemotherapy, *n* (%)								
No	403	(92.6)	48	(88.9)	83	(98.8)	272	(91.6)
Yes	32	(7.4)	6	(11.1)	1	(1.2)	25	(8.4)

The proportions of patients with a history of NMIBC or prior BCG therapy were lower in those with cT1 disease than in those with different clinical staging, and a higher proportion underwent RC at initial presentation. The prevalence of histological subtypes was also higher in the cT1 group. Furthermore, while very few patients with Ta or Tis disease received NAC, 17.2% of the patients with cT1 disease underwent NAC. National insurance coverage for robot-assisted RC has been approved in Japan since April 2018, and the proportion of robot-assisted procedures remained relatively low at approximately 13.1%. Standard or extended LND was performed in 83.9% of patients with available LND data. The median number of LNs removed was 6.5 (IQR, 3–8) for those who underwent limited dissection, 16 (IQR, 10–22) for those who received standard dissection, and 21 (IQR, 15–28.2) for those requiring extended dissection. Adjuvant chemotherapy was administered in 8.4% of cases.

### Overall oncological outcomes

Pathological MIBC and LN metastasis were observed in 101 (23.2%) and 28 (6.4%) patients, respectively. Upstaging to pT2 or pN+ disease occurred in 16/54 (29.6%) of patients with cTa, 8/84 (9.5%) of patients with cTis, and 81/297 (27.3%) of patients with cT1 disease.

**Fig. 2:**
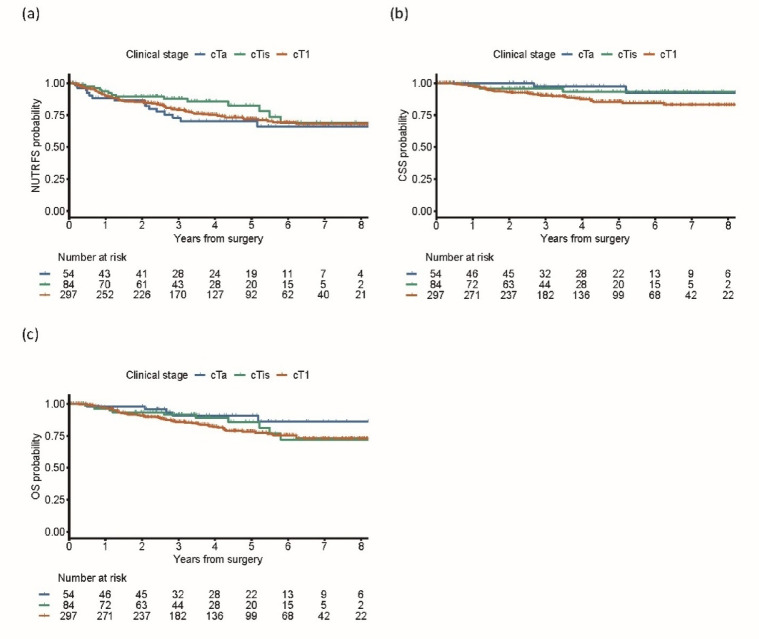
Overall survival outcomes of RC for NMIBC. Kaplan–Meier curves are shown for NUTRFS (**a**), CSS (**b**), and OS (**c**), stratified by clinical T stages. Deaths from any cause occurred in five patients with cTa disease, in 11 with cTis disease, and in 55 with cT1 disease. Cancer-specific mortality occurred in two patients with cTa disease, in four with cTis disease, and in 35 with cT1 disease. Non-urinary tract recurrence occurred in 15 patients with cTa, in 14 with cTis, and in 73 with cT1 disease. RC, radical cystectomy; NMIBC, non-muscle invasive bladder cancer; NUTRFS, non-urinary tract recurrence-free survival; CSS, cancer-specific survival; OS, overall survival.

The median follow-up period was 44.4 months (IQR, 25.9–68.7 months), during which 71 (16.3%) patients died. The 3- and 5-year NUTRFS rates were 72.9% (95% CI [61.2–86.8]) and 70.3% [58.2–84.9] for cTa, 87.7% [80.5–95.7] and 82.4% [72.8–93.3] for cTis, and 79.1% [74.3–84.2] and 71.8% [66.2–78.0] for cT1 disease, respectively (Fig. 2a). The corresponding CSS rates were 97.4% (95% CI [92.4–100]) and 97.4% [92.4–100] for cTa, 95.9% [91.5–100] and 93.3% [86.9–100] for cTis, and 90.3% [86.7–94.0] and 85.4% [80.7–90.3] for cT1, respectively (Fig. 2b). The corresponding OS rates were 90.6% (95% CI [82.2–99.9]) and 90.6% [82.2–99.9] for cTa, 91.6% [85.3–98.3] and 85.8% [76.4–96.4] for cTis, and 85.8% [81.6–90.2] and 78.1% [72.7–83.9] for cT1, respectively (Fig. 2c).

“Adequate BCG” was defined as at least five induction instillations plus two maintenance instillations, or seven or more induction instillations. BCG-unresponsive disease was defined as persistent high-grade bladder cancer despite adequate BCG or recurrence of high-grade disease within 1 year after adequate BCG. The cohort included 245 BCG-naïve, 77 BCG-unresponsive, 75 other BCG-failure, and 8 BCG-intolerant patients; one patient with unknown status was excluded. The latter two groups were combined as BCG failure/intolerant. Survival outcomes were compared among BCG-naïve, BCG-unresponsive, and BCG failure/intolerant groups, with no significant differences in OS, CSS, or NUTR (Supplemental Fig. 1). Upstaging occurred in 69/245 (28.2%) BCG-naïve, 14/77 (18.2%) BCG-unresponsive, and 19/83 (22.9%) BCG failure/intolerant patients.

The detailed distribution of NUTR by disease stage was as follows: for cT1 disease, 11 patients exhibited intrapelvic recurrence, 27 experienced distant recurrence, 13 had combined recurrence, and one patient’s status was unknown. For cTa disease, five patients exhibited intrapelvic recurrence and seven experienced distant recurrence. For cTis disease, two patients had intrapelvic recurrence and five demonstrated distant recurrence.

### Risk factors associated with poor survival outcomes in cTa and cT1 disease

Perioperative factors associated with poor prognosis after RC in patients with cTa and cT1 disease were explored. Among the 351 patients included in this analysis, 54 had cTa and 297 had cT1 disease, and 37 cancer-specific deaths occurred during the follow-up period (median, 45.9 months; IQR, 26.1–70.3 months).

**Fig. 3:**
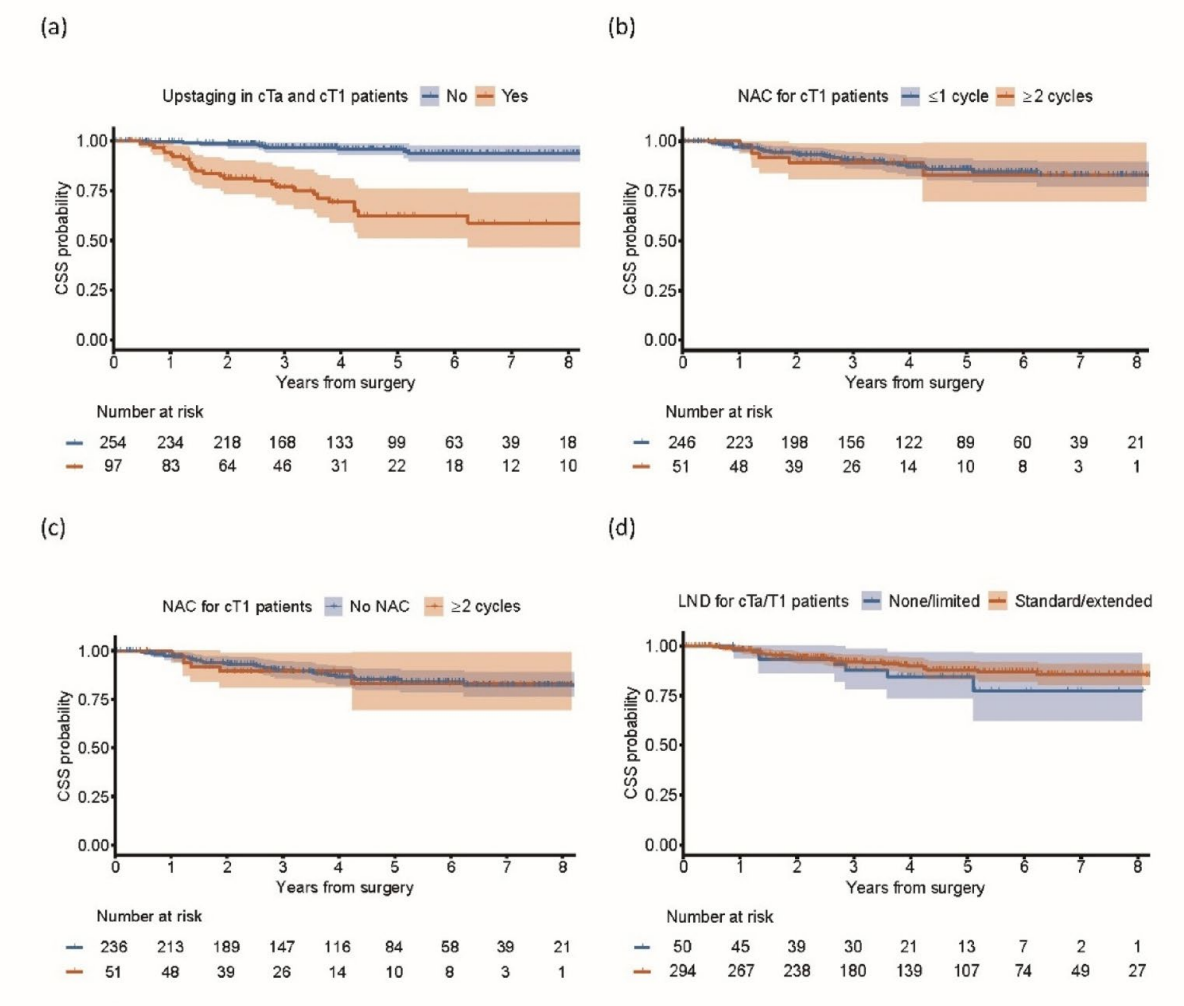
The impact of upstaging and the effect of NAC and LND on CSS after RC for cTa and cT1 disease. (**a**) Kaplan–Meier curves for CSS in patients with cTa and cT1 disease stratified by the presence of upstaging at the time of RC. Cancer-specific mortality occurred in 10 patients who did not experience upstaging and in 27 who did experience upstaging. (**b**) Kaplan–Meier curves for CSS in patients with cT1 disease stratified by NAC administration, comparing those who received ≤ 1 cycle with those who received ≥ 2 cycles. Cancer-specific mortality occurred in 29 patients who received insufficient NAC and in six who received sufficient NAC. (**c**) Sensitivity analysis comparing patients who received ≥ 2 cycles of NAC with those who received no NAC. (**d**) Kaplan–Meier curves for CSS in patients with cTa and cT1 disease stratified according to the extent of LND, both in the unadjusted cohorts. Cancer-specific mortality occurred in seven patients who underwent no/limited LND and in 30 who received standard/extended LND. NAC, neoadjuvant chemotherapy; LND, lymph node dissection; CSS, cancer-specific survival; RC, radical cystectomy.

In the univariable Cox proportional hazards analysis, age, ECOG-PS, calendar year of RC, upstaging to ≥pT2 or pN+ disease, and adjuvant chemotherapy were significantly associated with cancer-specific mortality (Table 2). Multivariable Cox proportional hazards analysis was subsequently performed, which included factors with a p-value < 0.1 in the univariable analysis and variables considered to be potentially associated with cancer-specific mortality. In the multivariable analysis, age and clinical T stage were marginally but significantly associated with poorer CSS; however, the association of calendar year of RC with CSS was attenuated and no longer statistically significant. Neither NAC nor LND was associated with an improvement in CSS (Table 2). Notably, upstaging was strongly associated with worse CSS (HR = 6.53 [95% CI, 3.02–14.8], log-rank test, *p* < 0.001, Table 2; Fig. 3a). The 3- and 5-year CSS rates were 96.6% (95% CI [94.2–99.1]) and 95.9% [93.2–98.8] for patients without upstaging, and 76.8% [68.1–86.7] and 62.5% [51.3–76.0] for those with upstaging, respectively. The results obtained from the multiply imputed datasets confirmed the findings of the complete case analysis (Supplemental Table 1).

**Table 2:** Cox proportional hazards analysis of risk factors associated with cancer-specific mortality in patients with cTa and cT1 bladder cancer based on the complete case dataset.

Variable	Univariable analysis		Multivariable analysis	
	HR (95% CI)	*P* value	HR (95% CI)	*P* value
Age (per 10 year increase)	1.61 (1.04–2.48)	**0.032**	1.73 (1.09–2.74)	**0.022**
Sex, male vs. female	1.06 (0.41–2.72)	0.9		
ECOG-PS, >=2 vs. 0–1	3.88 (1.18–12.73)	**0.025**	2.10 (0.61–7.56)	0.3
Smoking history	0.79 (0.37–1.66)	0.5		
History of NMIBC	1.32 (0.68–2.54)	0.4		
History of BCG	0.58 (0.25–1.33)	0.2		
Tumour multiplicity, multiple vs. solitary	2.06 (0.80–5.34)	0.1		
Size of tumours, >=3 cm vs. <3 cm	1.52 (0.75–3.07)	0.2		
Clinical T stage, cT1 vs. cTa	3.28 (0.79–13.62)	0.1	4.48 (1.11–20.40)	**0.043**
Concomitant CIS (TUR and/or RC)	0.74 (0.39–1.42)	0.4		
NAC, >=2 cylcles vs. 0–1 cycle	1.18 (0.49–2.84)	0.7	2.44 (0.85–5.56)	0.066
Calendar year of RC (per 1 year increase)	0.74 (0.60–0.91)	**0.004**	0.81 (0.61–0.92)	0.064
Surgical technique				
Laparoscopic vs. open	0.42 (0.18–1.02)	0.054	0.48 (0.15–0.90)	0.1
Robotic vs. open	0.22 (0.03–1.59)	0.1	0.40 (0.03–1.69)	0.4
LND, standard/extended vs. none/limited	0.66 (0.29–1.50)	0.3		
Upstaging to > = pT2 or pN+ disease	9.00 (4.35–18.63)	**< 0.001**	6.53 (3.02–14.81)	**< 0.001**
Histological subtype (TUR and/or RC)	1.01 (0.44–2.54)	0.9		
Adjuvant chemotherapy	4.25 (2.06–8.79)	**< 0.001**	1.97 (0.91–4.92)	0.12

Significant values are in bold.

### Effect of NAC on survival outcomes in cTa and cT1 disease

The effect of NAC in patients with cTa and cT1 disease was further investigated in greater detail. Only five patients with cTa disease underwent ≥ 2 cycles of NAC; therefore, the impact of NAC could only be evaluated in patients with cT1 disease, 82 (25.5%) of whom underwent NAC.

In patients with cT1 disease, substantial overlap of the Kaplan–Meier curves was observed for those who received ≤ 1 cycle of NAC and those who received ≥ 2 cycles (HR = 1.16 [0.48–2.79], log-rank test, *p* = 0.8, Fig. 3b). Similar results were observed in a sensitivity analysis excluding patients who received only one cycle of NAC and comparing those who received ≥ 2 cycles with those who received no NAC (HR = 1.10 [0.46–2.66], log-rank test, *p* = 0.8).

After applying the IPTW (Supplemental Fig. 2a), there was no significant difference in the CSS rates between those two groups (HR = 1.46 [0.49–4.36], log-rank test, *p* = 0.8; Supplemental Fig. 2b). Similar results were observed for the IPTW analysis of the multiply imputed datasets (HR = 1.26 [0.48–3.32], *p* = 0.6; Supplemental Fig. 2c). These findings, which were consistent with the Cox proportional hazards model results, suggest that NAC does not confer a survival benefit in patients with cT1 bladder cancer.

### Effect of LND on survival outcomes in cTa and cT1 disease

The effect of LND in patients with cTa and cT1 disease was also subsequently investigated in greater detail. In the unadjusted cohort, there was no significant difference in CSS between those who underwent no LND or limited LND and those who received standard or extended LND (HR = 0.66 [0.29–1.50], log-rank test, *p* = 0.32; Fig. 3d). Similarly, no significant difference in CSS was observed according to the extent of LND in the IPTW analysis adjusted for baseline characteristics (HR = 0.99 [0.26–3.71], log-rank test, *p* = 0.4; Supplemental Fig. 3a and b), consistent with the findings of the Cox proportional hazards model. IPTW was also performed using the multiply imputed datasets; however, patients who did not undergo LND were markedly older than those who did, and the SMD for age remained high despite the adjustment; therefore, that analysis was not completed. Instead, propensity score matching was conducted as a sensitivity analysis, which confirmed that LND was not associated with a significant improvement in CSS (HR = 0.95 [0.13–6.71], log-rank test, *p* > 0.9; Supplemental Table 2, Supplemental Fig. 3c).

In the cTa and cT1 cohorts, pathological LN metastases were identified in 1/25 (4.0%) patients who underwent limited LND, in 14/165 (8.5%) who underwent standard LND, and in 11/129 (8.5%) who underwent extended LND, suggesting that LND may provide diagnostic benefits. Adjuvant chemotherapy was administered in 19/27 (70.4%) patients with pN+ disease and in only 12 (3.7%) of the 324 patients with pN- or pNX disease.

### Upstaging-related risk factors in cTa and cT1 disease

The findings of the previous analysis of risk factors associated with cancer-specific mortality suggested that the ability to predict upstaging to ≥pT2 or pN+ disease prior to RC based on preoperative factors could be of clinical value. Therefore, logistic regression analyses were conducted to identify potential predictive factors among 351 patients with cTa and cT1 disease. Ninety-seven patients (27.6%) experienced pathological upstaging. In the univariable analysis, tumour size (≥ 3 cm) was marginally but significantly associated with an increased upstaging risk, whereas the presence of histological subtypes was marginally but significantly associated with a *decreased* risk of upstaging (Table 3). Multivariable analysis was subsequently performed, which included factors from the univariable analysis with a p-value < 0.1 and variables considered to be potentially associated with upstaging, such as the clinical T stage, concomitant CIS, and NAC administration. Tumour size (≥ 3 cm) alone was significantly associated with an increased risk of upstaging (OR = 1.90 [1.06–3.40], *p* = 0.03; Table 3). The results obtained from the multiply imputed datasets confirmed the findings of the complete case analysis; however, the presence of histological subtypes was marginally but significantly associated with a *decreased* risk of upstaging (Supplemental Table 3).

**Table 3:** Logistic regression analysis of preoperative risk factors associated with pathological upstaging in patients with cTa and cT1 bladder cancer based on complete case dataset.

Variable	Univariable analysis		Multivariable analysis	
	OR (95% CI)	*P* value	OR (95% CI)	*P* value
Age (per 10 year increase)	1.24 (0.94–1.64)	0.1		
Sex, male vs. female	0.79 (0.41–1.53)	0.5		
ECOG-PS, >=2 vs. 0–1	2.52 (0.71–8.92)	0.2		
Smoking history	0.70 (0.40–1.22)	0.2		
History of NMIBC	1.07 (0.66–1.73)	0.8		
History of BCG	0.79 (0.47–1.34)	0.4		
Tumour multiplicity, multiple vs. solitary	1.15 (0.67–2.00)	0.6		
Size of tumours, >=3 cm vs. <3 cm	1.86 (1.09–3.16)	**0.022**	1.90 (1.06–3.40)	**0.031**
Clinical T stage, cT1 vs. cTa	0.94 (0.69–1.30)	0.7	0.86 (0.59–1.25)	0.4
Concomitant CIS (TUR)	0.69 (0.41–1.17)	0.2	0.74 (0.41–1.36)	0.3
Histological subtype (TUR)	0.38 (0.15–0.92)	**0.033**	0.40 (0.16–1.01)	0.053
NAC, >=2 cylcles vs. 0–1 cycle	0.95 (0.50–1.81)	0.9	0.65 (0.31–1.37)	0.3

Significant values are in bold.

In response to the counterintuitive finding that the histological subtype was associated with a *decreased* risk of upstaging, stratified and sensitivity analyses were performed in which variant histology was excluded, based on the complete-case dataset. Although no statistically significant associations were observed, older age and tumour size (≥ 3 cm) showed a trend toward an increased risk of upstaging (Supplemental Table 4).

### Additional analysis of cTis disease

Patients with cTis disease were not included in the analysis of risk factors associated with cancer-specific mortality, as important covariates such as tumour size, multiplicity, and concomitant CIS could not be applied. Therefore, the impact of upstaging to ≥pT2 or pN+ disease and the effects of NAC and LND on CSS in patients with cTis were investigated separately.

Upstaging was significantly associated with poorer CSS also in patients with cTis disease (HR = 7.48 [1.04–53.7], log-rank test, *p* = 0.02) (Supplemental Fig. 4a). Only six patients received ≥ 2 cycles of NAC; therefore, the statistical significance of receiving sufficient NAC in patients with CIS was not evaluated. Twenty-two patients underwent either no LND or limited LND, whereas 62 patients received either standard or extended LND; the 5-year CSS rates were 90.0% and 94.3% in the former and latter groups, respectively, with no significant intergroup difference (HR = 1.16 [0.12–11.1], log-rank test, *p* = 0.9) (Supplemental Fig. 4b). LN metastasis was not observed in any of the six patients who underwent limited LND, whereas only one of the 62 (1.6%) patients who underwent standard or extended LND was classified as pN+, suggesting that LND had limited diagnostic value in this population.

## Discussion

In this study based on a multi-institutional database from Japan, the oncological outcomes of RC for NMIBC were generally favourable, which is consistent with previous reports^13^. Survival outcomes tended to be worse in patients with cT1 disease than in those with cTa or cTis disease, reflecting the overall trend. However, patients who experienced pathological upstaging to ≥pT2 or pN+ disease exhibited suboptimal survival outcomes, with a 3-year CSS rate of approximately 75%. These findings align with recent data reported by Scilipoti et al.^15^ and are comparable to outcomes observed in patients undergoing RC for MIBC^15^.

Previous studies that have investigated the prognostic impact of concomitant CIS in patients who underwent RC have reported conflicting results, with some identifying it as a factor associated with poor prognosis^16^ and others reporting no significant impact on outcomes^17^. However, their cohorts primarily consisted of patients with MIBC, and the prognostic impact of concomitant CIS in patients with NMIBC undergoing RC has remained unclear. In the present study, concomitant CIS was not associated with a worse prognosis in patients with cTa and cT1 disease; in fact, both the HR for cancer-specific mortality and the OR for upstaging were less than 1, although the difference was not statistically significant. CIS has been identified as a key factor in the classification of high-risk and very high-risk NMIBC according to clinical guidelines^4,5,7^. In cases with such high-risk features, patients may be more likely to undergo early RC, potentially contributing to lower rates of upstaging and cancer-specific mortality.

Although some studies have demonstrated a potential survival benefit of NAC in patients with T1 disease and clinical node involvement^18,19^, there remains no consensus regarding the clinical significance of NAC in the context of RC for NMIBC. The proportion of patients who received NAC in the present study was comparable to that in a large U.S. database study^8^. In Japan, NAC is generally not administered to treat NMIBC; however, in certain situations, individual clinicians or institutional policies may consider NAC to be appropriate, such as in instances involving prolonged waiting times prior to surgery in patients with large tumours that cannot be completely resected and when there may be understaging concerns despite the imaging findings not being clearly suggestive of MIBC or indicating the presence of lymphovascular invasion. Unfortunately, the database did not provide information on the specific indications for the administration of NAC; therefore, the detailed rationale underlying its use remains unknown. NAC administration in patients with cT1 disease resulted in no significant improvement in CSS, and it did not significantly reduce the risk of pathological upstaging in either the univariable or multivariable analyses, suggesting a lack of therapeutic benefits of NAC in the context of RC for NMIBC. A rational therapeutic strategy would be to consider administering postoperative adjuvant therapy in cases in which pathological upstaging is observed.

Among the 428 patients for whom LND data were available, 356 (83.2%) underwent standard or extended LND, although there is currently no consensus on what constitutes “routine” LND at the time of RC for NMIBC. This suggests that surgeons generally consider both bladder excision and LND as integral components of the RC procedure. However, no association was observed between LND and improved CSS. Of the patients who underwent standard or extended LND, 7.3% had a pN+ classification, which is consistent with previously reported ranges (6.6–16.2%) ^20–22^ and supports the validity of the present findings. Although its therapeutic benefits remain unclear, LND may have diagnostic value in guiding clinical decision-making regarding postoperative adjuvant therapy. However, it was not possible to determine whether the administration of adjuvant chemotherapy to patients with pN+ disease contributed to improved survival outcomes in this study based on the available data.

Pathological upstaging was by far the most impactful risk factor associated with cancer-specific mortality, underscoring the importance of being able to predict upstaging preoperatively. The upstaging rate was 24.1% in the entire cohort, which is also consistent with previously reported values ranging from 22 to 46% ^23–25^. The frequency of upstaging was higher for cTa tumours than it was for cTis tumours, and the rate was comparable to that observed in cT1 disease. This suggests that in cases of NMIBC involving tumour formation, staging errors may occur as a result of incomplete TURBT or inadequate preoperative imaging, highlighting the inherent difficulty of accurately determining clinical staging in NMIBC. Several factors have been identified as potential predictors of upstaging, including the presence of lymphovascular invasion and histological subtypes, tumour multiplicity, and the number of TURBT procedures performed prior to RC ^26–28^. However, the findings have been inconsistent across studies, and the sample sizes have generally been small; therefore, no established predictive factors have been identified to date. Although a tumour size ≥ 3 cm was identified as the only significant preoperative predictor of upstaging in the main multivariable model, its association with that outcome became non-significant in the sensitivity analyses, suggesting that the effect may be modest and sensitive to model specifications. Unexpectedly, the presence of histological subtypes was associated with a lower risk of upstaging, contrary to previous reports^26^. The presence of histological subtypes is a key determinant in the very high-risk classification of NMIBC^4,5,7^. Patients exhibiting histological subtypes may preferentially undergo earlier RC, which could potentially help reduce the likelihood of upstaging. Overall, the findings of the present study highlight the difficulty of identifying factors to predict upstaging prior to RC.

Although this study analysed data derived from a multi-institutional database, it has some inherent limitations, including its retrospective design and the relatively small sample size, reflecting the limited indications for RC in NMIBC. One of the major limitations was the fact that data were missing for multiple variables, which hindered the ability to minimise bias in the analyses; however, the results of the complete case analysis were validated using datasets in which missing values were imputed through a multiple imputation method. Although IPTW was employed to balance the baseline characteristics between groups, perfect balance was not achieved, and the potential influence of unmeasured confounding factors remains a concern. Another limitation was the absence of a central review of imaging and pathological assessments, which may have led to variability in the diagnostic accuracy. Furthermore, data on the clinical application of magnetic resonance imaging (MRI) and VI-RADS criteria^29^ for clinical T staging, the use and indications of random biopsy for CIS detection, and the implementation of a second transurethral resection were unavailable, limiting the ability to fully evaluate the accuracy of the preoperative diagnoses. This limitation may also affect the external validity and generalisability of the present findings to other settings. In addition, the database did not include predefined criteria for RC in patients with NMIBC, and the specific clinical reasons for proceeding with RC were unavailable. More than half the cohort underwent RC without prior BCG therapy, despite recent reports indicating a shift toward bladder-preserving approaches, even among patients with very-high-risk NMIBC^14^. In the absence of clearly documented indications, it is difficult to determine the rationale for this practice pattern or to identify subgroups in whom immediate RC might have been deferred. Therefore, the findings should be interpreted with caution in the context of treatment decision-making. Lastly, although integrating molecular risk stratification with advanced imaging modalities and clinical variables could ultimately provide a more accurate and reliable staging framework, such approaches were not available in the present study. Despite these limitations, this study represents one of the largest cohorts of patients undergoing RC for NMIBC and provides valuable insights through statistically robust and comprehensive analyses.

Ultimately, the survival outcomes of RC for NMIBC were generally favourable. Neither NAC nor LND significantly improved the survival outcomes. Upstaging was the most important risk factor for cancer-specific mortality. While the ability to predict upstaging preoperatively would be of great clinical value, accurately identifying patients at risk of upstaging when making treatment-related decisions remains extremely challenging. Recently, several studies have attempted to predict upstaging using genomic, transcriptomic, and multi-omics data^30–32^; however, these emerging tools remain largely investigational, and any potential clinical utility requires rigorous validation based on well-designed prospective cohort studies. Further advances in this field are eagerly anticipated.

## Supplementary Information

Below is the link to the electronic supplementary material.

Supplementary Material 1

Supplementary Material 2

## Data Availability

Data sets generated during and/or analysed during the current study are available from the corresponding author on reasonable request.
