# Supplementary material for: Comprehensive analysis of oncological outcomes of radical cystectomy for non-muscle invasive bladder cancer
Source: Sci Rep. 2026 Apr 5;16:16338. doi: 10.1038/s41598-026-46649-w (PMC13212721; doi:10.1038/s41598-026-46649-w)
Supplement: Supplementary file 1 — Supplementary Material 1 [file 41598_2026_46649_MOESM1_ESM.docx]

**Supplemental tables**

| Table S1. Cox proportional hazards analysis of risk factors associated with cancer-specific mortality in patients with cTa and cT1 bladder cancer based on the multiply imputed datasets. | | | | | |
| --- | --- | --- | --- | --- | --- |
|  | **Univariable analysis** | |  | **Multivariable analysis** | |
| **Variable** | **HR (95% CI)** | ***P* value** |  | **HR (95% CI)** | ***P* value** |
| Age (per 10 year increase) | No missing values | |  | 1.72 (1.06–2.78) | **0.028** |
| Sex, male vs. female | No missing values | |  |  |  |
| ECOG-PS, >=2 vs. 0–1 | 4.11 (1.19–14.15) | **0.026** |  | 2.18 (0.58–8.14) | 0.2 |
| Smoking history | 0.79 (0.37–1.71) | 0.5 |  |  |  |
| History of NMIBC | 1.31 (0.66–2.60) | 0.4 |  |  |  |
| History of BCG | 0.57 (0.24–1.33) | 0.2 |  |  |  |
| Tumour multiplicity, multiple vs. solitary | 1.99 (0.75–5.31) | 0.2 |  |  |  |
| Size of tumours, >=3 cm vs. <3cm | 1.48 (0.71–3.10) | 0.3 |  |  |  |
| Clinical T stage, cT1 vs. cTa | No missing values | |  | 4.86 (1.06–22.19) | **0.042** |
| Concomitant CIS (TUR and/or RC) | No missing values | |  |  |  |
| NAC, >=2 cylcles vs. 0–1 cycle | No missing values | |  | 2.17 (0.81–5.83) | 0.1 |
| Calendar year of RC | 0.74 (0.60-0.91) | **0.006** |  | 0.74 (0.60–0.92) | **0.007** |
| LND, standard/extended vs. none/limited | 0.68 (0.29–1.61) | 0.4 |  |  |  |
| Upstaging to >=pT2 or pN+ disease | No missing values | |  | 7.08 (3.09–16.23) | **< 0.001** |
| Histological subtype (TUR and/or RC) | 1.06 (0.43–2.62) | 0.9 |  |  |  |
| Adjuvant chemotherapy | No missing values | |  | 2.13 (0.88–5.17) | 0.09 |

| Table S2. Baseline and perioperative characteristics of patients with cTa and cT1 bladder cancer by the extent of LND after PSM. | | | | | |
| --- | --- | --- | --- | --- | --- |
|  | **None/limited LND** | | **Standard/extended LND** | |  |
|  | **(*n* = 28)** | | **(*n* = 28)** | | ***P* value** |
| Age at RC, medial (IQR), y | 75 | (71;80) | 74 | (70;78) | 0.6 |
| Sex, *n* (%) |  |  |  |  | > 0.9 |
| Male | 22 | (78.6) | 22 | (78.6) |  |
| Female | 6 | (21.4) | 6 | (21.4) |  |
| BMI, median (IQR) | 22.2 | (20.3;24.8) | 23.9 | (20.7;25.3) | 0.3 |
| Smoking status, *n* (%) |  |  |  |  | 0.8 |
| None | 8 | (28.6) | 10 | (35.7) |  |
| Past/current | 20 | (71.4) | 18 | (64.3) |  |
| History of NMIBC, *n* (%) |  |  |  |  | 0.3 |
| No | 17 | (60.7) | 12 | (42.9) |  |
| Yes | 11 | (39.3) | 16 | (57.1) |  |
| History of BCG, *n* (%) |  |  |  |  | 0.4 |
| No | 19 | (67.9) | 15 | (53.6) |  |
| Yes | 9 | (32.1) | 13 | (46.4) |  |
| Tumour multiplicity, *n* (%) |  |  |  |  | > 0.9 |
| Single | 6 | (21.4) | 5 | (17.9) |  |
| Multiple | 22 | (78.6) | 23 | (82.1) |  |
| Size of tumours, *n* (%) |  |  |  |  | 0.8 |
| Less than 3 cm | 21 | (75.0) | 19 | (67.9) |  |
| 3 cm or larger | 7 | (25.0) | 9 | (32.1) |  |
| Concurrent CIS at TUR, *n* (%) |  |  |  |  | > 0.9 |
| No | 19 | (67.9) | 18 | (64.3) |  |
| Yes | 9 | (32.1) | 10 | (35.7) |  |

| Table S3. Logistic regression analysis of preoperative risk factors associated with pathological upstaging in patients with cTa and cT1 bladder cancer based on the multiply imputed datasets. | | | | | |
| --- | --- | --- | --- | --- | --- |
|  | **Univariable analysis** | |  | **Multivariable analysis** | |
| **Variable** | **OR (95% CI)** | ***P* value** |  | **OR (95% CI)** | ***P* value** |
| Age (per 10 year increase) | No missing values | |  |  |  |
| Sex, male vs. female | No missing values | |  |  |  |
| ECOG-PS, >=2 vs. 0–1 | 2.51 (0.71–8.93) | 0.2 |  |  |  |
| Smoking history | 0.75 (0.43–1.30) | 0.3 |  |  |  |
| History of NMIBC | 1.06 (0.65–1.71) | 0.8 |  |  |  |
| History of BCG | 0.79 (0.47–1.35) | 0.4 |  |  |  |
| Tumour multiplicity, multiple vs. solitary | 1.16 (0.67–2.02) | 0.6 |  |  |  |
| Size of tumours, >=3 cm vs. <3cm | 1.85 (1.09–3.14) | **0.023** |  | 1.93 (1.09–3.40) | **0.024** |
| Clinical T stage, cT1 vs. cTa | No missing values | |  | 1.00 (0.52–1.92) | > 0.9 |
| Concomitant CIS (TUR) | 0.71 (0.42–1.19) | 0.2 |  | 0.83 (0.48–1.44) | 0.5 |
| Histological subtype (TUR) | 0.38 (0.16–0.94) | **0.037** |  | 0.36 (0.14–0.90) | **0.029** |
| NAC, >=2 cylcles vs. 0–1 cycle | No missing values | |  | 0.80 (0.41–1.59) | 0.5 |

| Table S4. Logistic regression analysis of preoperative risk factors associated with pathological upstaging in patients with cTa and cT1 bladder cancer excluding patients with histlogical subtype based on complete case dataset. | | | | | |
| --- | --- | --- | --- | --- | --- |
|  | **Univariable analysis** | |  | **Multivariable analysis** | |
| **Variable** | **OR (95% CI)** | ***P* value** |  | **OR (95% CI)** | ***P* value** |
| Age (per 10 year increase) | 1.31 (0.97–1.78) | 0.080 |  | 1.41 (0.97-2.05) | 0.070 |
| Sex, male vs. female | 0.78 (0.39–1.57) | 0.5 |  |  |  |
| ECOG-PS, >=2 vs. 0–1 | 1.85 (0.48–7.09) | 0.4 |  |  |  |
| Smoking history | 0.69 (0.38–1.23) | 0.2 |  |  |  |
| History of NMIBC | 1.06 (0.64–1.75) | 0.8 |  |  |  |
| History of BCG | 0.71 (0.41–1.23) | 0.2 |  |  |  |
| Tumour multiplicity, multiple vs. solitary | 1.14 (0.64–2.03) | 0.7 |  |  |  |
| Size of tumours, >=3 cm vs. <3cm | 1.97 (1.11–3.50) | **0.020** |  | 1.74 (0.94–3.24) | 0.080 |
| Clinical T stage, cT1 vs. cTa | 0.93 (0.48–1.79) | 0.8 |  | 1.22 (0.56–2.66) | 0.6 |
| Concomitant CIS (TUR) | 0.71 (0.41–1.23) | 0.2 |  | 0.79 (0.41–1.49) | 0.5 |
| NAC, >=2 cylcles vs. 0–1 cycle | 1.02 (0.52–2.01) | > 0.9 |  | 0.79 (0.35–1.77) | 0.6 |
