# Supplementary material for: Comprehensive analysis of oncological outcomes of radical cystectomy for non-muscle invasive bladder cancer
Source: Sci Rep. 2026 Apr 5;16:16338. doi: 10.1038/s41598-026-46649-w (PMC13212721; doi:10.1038/s41598-026-46649-w)
Supplement: Supplementary file 2 — Supplementary Material 2 [file 41598_2026_46649_MOESM2_ESM.pdf]

Supplementary figure 1

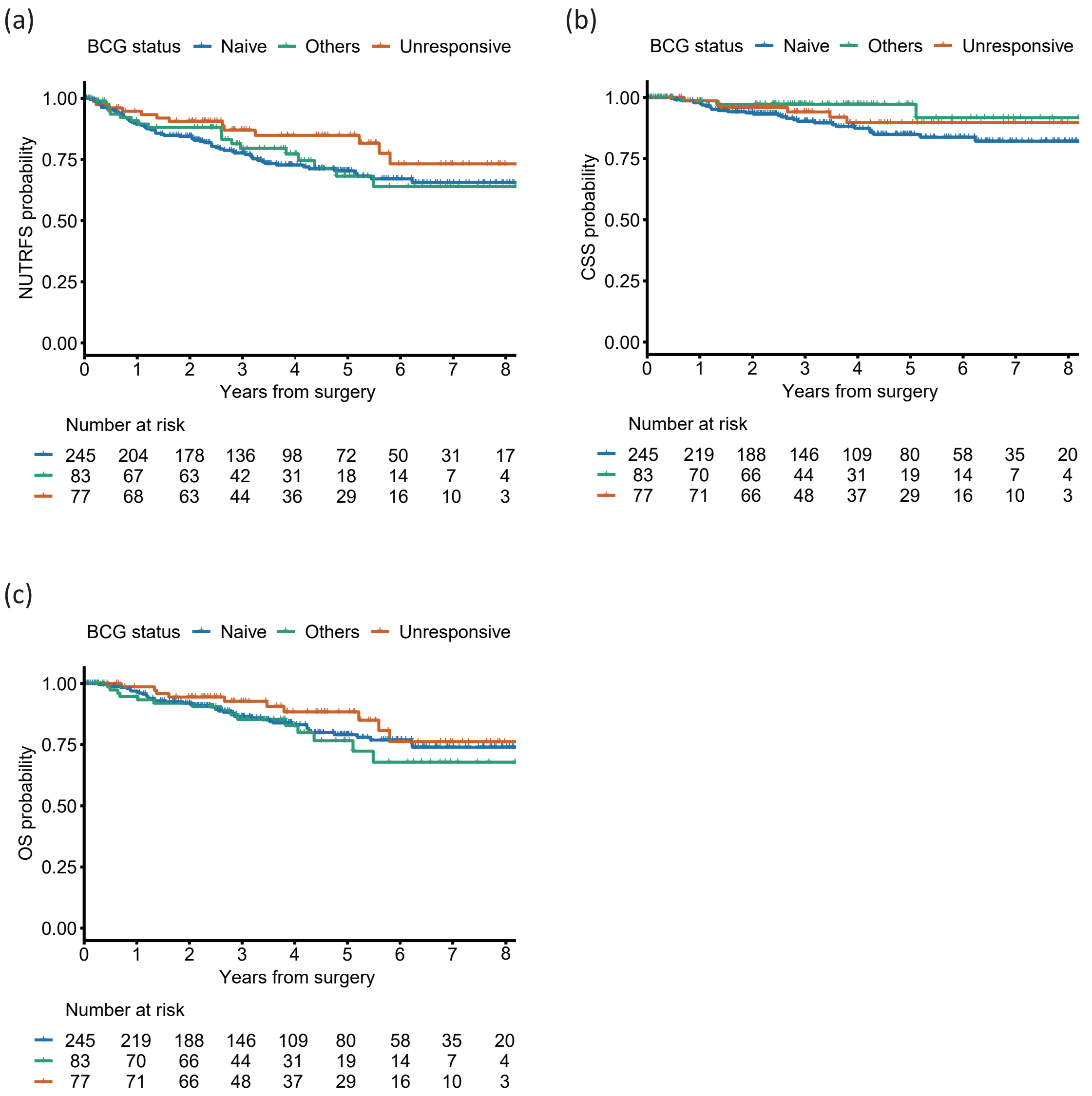

Figure S1. Overall survival outcomes of RC for NMIBC stratified by BCG status. Kaplan–Meier curves are shown for NUTRFS (a), CSS (b), and OS (c). RC, radical cystectomy; NMIBC, non-muscle invasive bladder cancer; BCG, Bacillus Calmette-Guérin, NUTRFS, non-urinary tract recurrence-free survival; CSS, cancer-specific survival; OS, overall survival.

Supplementary figure 2

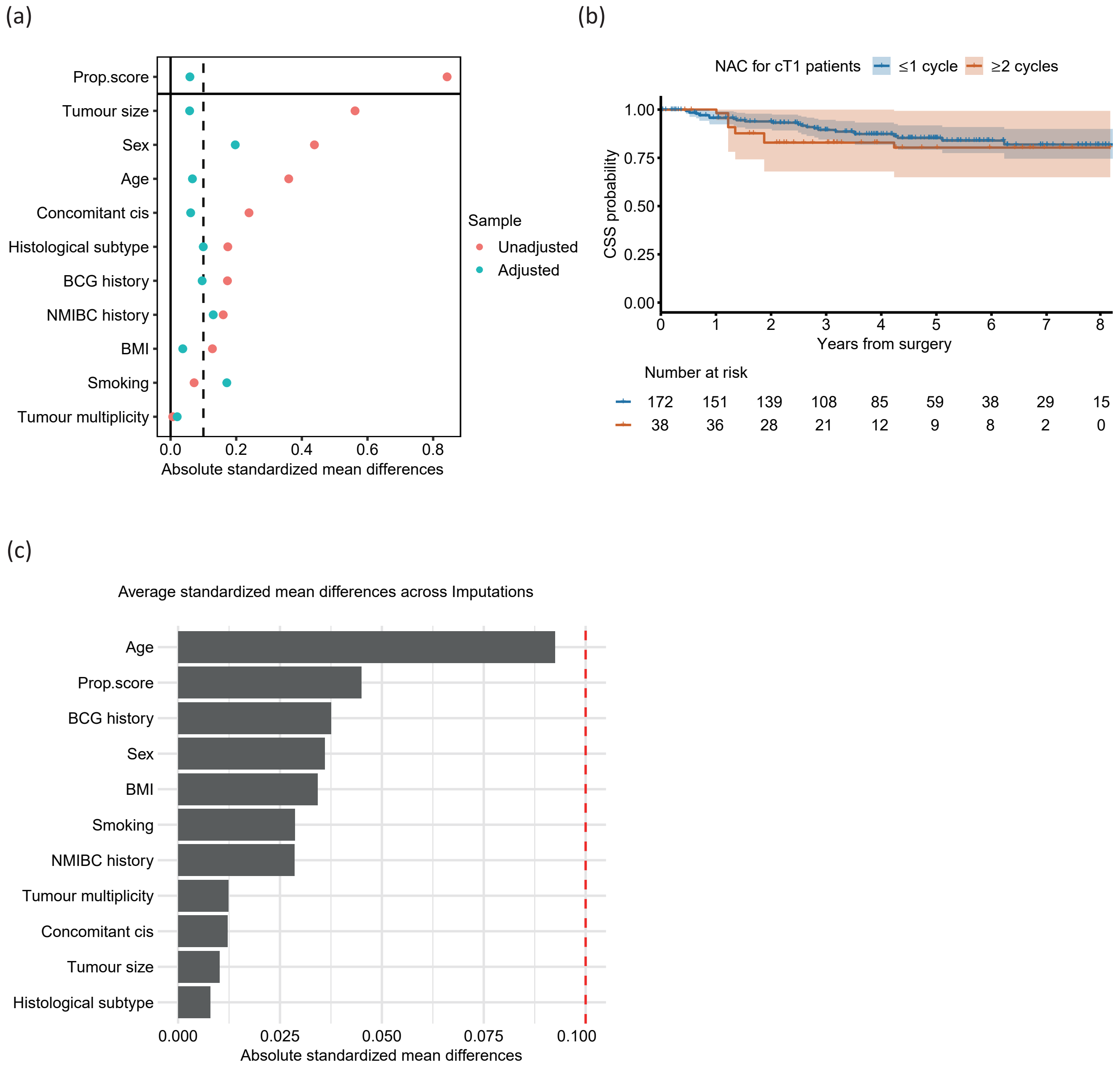

Figure S2. (a) SMDs for the balancing of covariates before and after IPTW in patients with cT1 disease, weighted according to the administration of NAC. IPTW improved the covariate balance between groups. Among the 10 covariates, the adjusted SMDs for one covariate slightly exceeded the conventional threshold of 0.1, and those for two others were approximately 0.2. (b) Kaplan–Meier curves for CSS in patients with cT1 disease, stratified by the administration of NAC in the IPTW-adjusted cohort. (c) Average SMDs across multiply imputed datasets showing the balance of covariates after IPTW in patients with cT1 disease, weighted according to the administration of NAC. SMD, standardised mean difference; IPTW, inverse probability of treatment weighting; NAC, neoadjuvant chemotherapy; CSS, cancer-specific survival.

Supplementary figure 3

(a)

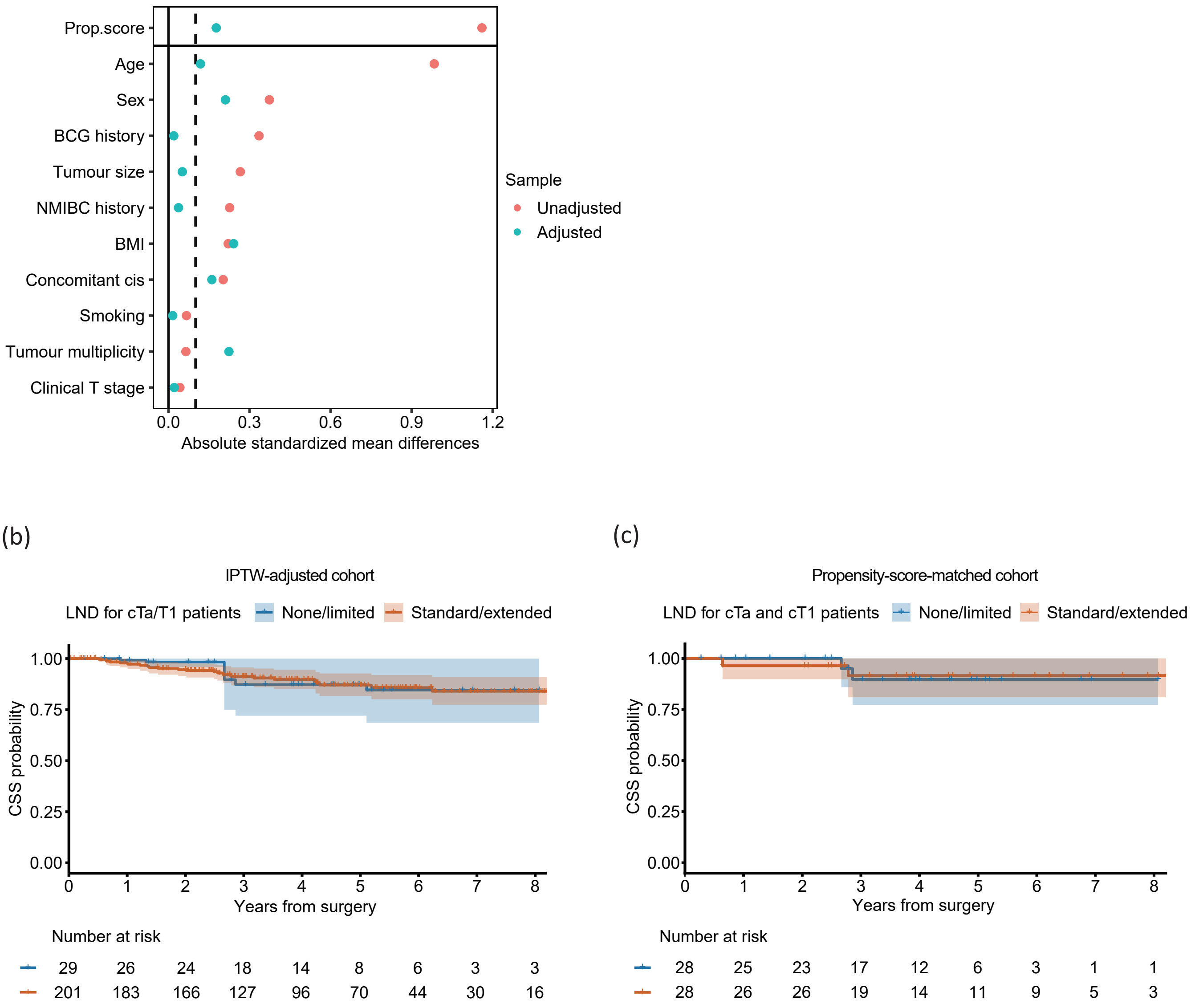

Figure S3. (a) SMDs for the balancing of covariates before and after IPTW in patients with cTa or cT1 disease, weighted according to the extent of LND. IPTW improved the covariate balance between groups. Among the 10 covariates, the adjusted SMDs for two covariates slightly exceeded the conventional threshold of 0.1, and those for three others were approximately 0.2. (b) Kaplan–Meier curves for CSS in patients with cTa or cT1 disease stratified by the extent of LND in the IPTW-adjusted cohort. (c) Kaplan–Meier curves for CSS in patients with cTa or cT1 disease stratified by the extent of LND in the propensity-score-matched cohort. Cancer-specific mortality occurred in two patients in both none/limited LND group and standard/extended LND group. SMD, standardised mean difference; IPTW, inverse probability of treatment weighting; LND, lymph node dissection; CSS, cancer-specific survival.

Supplementary figure 4

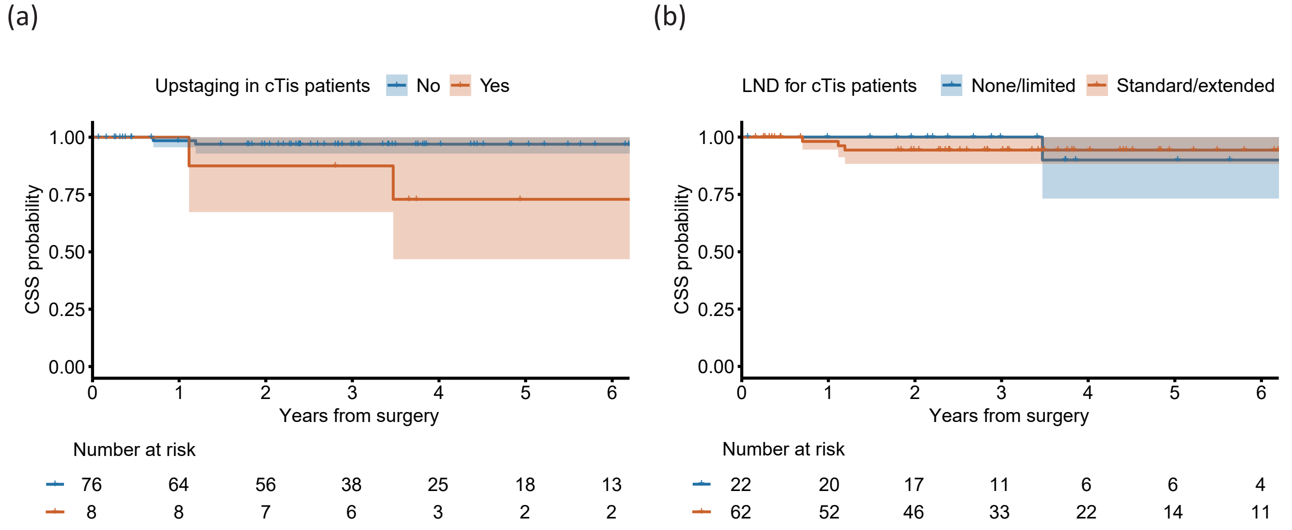

Figure S4. The impact of upstaging and the effect of LND on CSS after RC for cTis disease. (a) Kaplan–Meier curves for CSS stratified by the presence of upstaging at the time of RC. Cancer-specific mortality occurred in two patients in both non-upstaging group and upstaging group. (b) Kaplan–Meier curves for CSS stratified by the extent of LND. Cancer-specific mortality occurred in one patients with none/limited LND and three patients with standard/extended LND. LND, lymph node dissection; CSS, cancer-specific survival; RC, radical cystectomy.
